# Reasons for Facebook Usage: Data From 46 Countries

**DOI:** 10.3389/fpsyg.2020.00711

**Published:** 2020-04-30

**Authors:** Marta Kowal, Piotr Sorokowski, Agnieszka Sorokowska, Małgorzata Dobrowolska, Katarzyna Pisanski, Anna Oleszkiewicz, Toivo Aavik, Grace Akello, Charlotte Alm, Naumana Amjad, Afifa Anjum, Kelly Asao, Chiemezie S. Atama, Derya Atamtürk Duyar, Richard Ayebare, Mons Bendixen, Aicha Bensafia, Boris Bizumic, Mahmoud Boussena, David M. Buss, Marina Butovskaya, Seda Can, Katarzyna Cantarero, Antonin Carrier, Hakan Cetinkaya, Daniel Conroy-Beam, Marco A. C. Varella, Rosa M. Cueto, Marcin Czub, Daria Dronova, Seda Dural, Izzet Duyar, Berna Ertugrul, Agustín Espinosa, Ignacio Estevan, Carla S. Esteves, Tomasz Frackowiak, Jorge Contreras-Graduño, Farida Guemaz, Ivana Hromatko, Chin-Ming Hui, Iskra Herak, Jas L. Jaafar, Feng Jiang, Konstantinos Kafetsios, Tina Kavcic, Leif Edward Ottesen Kennair, Nicolas Kervyn, Nils C. Köbis, András Láng, Georgina R. Lennard, Ernesto León, Torun Lindholm, Giulia Lopez, Mohammad Madallh Alhabahba, Alvaro Mailhos, Zoi Manesi, Rocío Martínez, Sarah L. McKerchar, Norbert Meskó, Girishwar Misra, Hoang Moc Lan, Conal Monaghan, Emanuel C. Mora, Alba Moya Garófano, Bojan Musil, Jean C. Natividade, George Nizharadze, Elisabeth Oberzaucher, Mohd S. Omar Fauzee, Ike E. Onyishi, Baris Özener, Ariela F. Pagani, Vilmante Pakalniskiene, Miriam Parise, Farid Pazhoohi, Mariia Perun, Annette Pisanski, Nejc Plohl, Camelia Popa, Pavol Prokop, Muhammad Rizwan, Mario Sainz, Svjetlana Salkičević, Ruta Sargautyte, Susanne Schmehl, Oksana Senyk, Rizwana Shaikh, Shivantika Sharad, Franco Simonetti, Meri Tadinac, Truong Thi Khanh Ha, Trinh Thi Linh, Karina Ugalde González, Nguyen Van Luot, Christin-Melanie Vauclair, Luis D. Vega, Gyesook Yoo, Stanislava Yordanova Stoyanova, Zainab F. Zadeh, Maja Zupančič

**Affiliations:** ^1^University of Wrocław, Wrocław, Poland; ^2^Silesian University of Technology, Gliwice, Poland; ^3^Department of Otorhinolaryngology, Smell and Taste Clinic, Carl Gustav Carus Medical School, TU Dresden, Dresden, Germany; ^4^University of Tartu, Tartu, Estonia; ^5^Gulu University, Gulu, Uganda; ^6^Stockholm University, Stockholm, Sweden; ^7^University of the Punjab, Lahore, Pakistan; ^8^Westminster College, Salt Lake City, UT, United States; ^9^University of Nigeria, Nsukka, Nigeria; ^10^Sivas Cumhuriyet University, Sivas, Turkey; ^11^Grace Medical Clinic, Kampala, Uganda; ^12^Norwegian University of Science and Technology (NTNU), Trondheim, Norway; ^13^University Algiers2, Algiers, Algeria; ^14^Australian National University, Canberra, ACT, Australia; ^15^University of Texas at Austin, Austin, TX, United States; ^16^Institute of Ethnology and Anthropology, Moscow, Russia; ^17^İzmir University of Economics, İzmir, Turkey; ^18^SWPS University of Social Sciences and Humanities, Wrocław, Poland; ^19^University of Bordeaux, Bordeaux, France; ^20^Ankara University, Ankara, Turkey; ^21^University of California, Santa Barbara, Santa Barbara, CA, United States; ^22^University of São Paulo, São Paulo, Brazil; ^23^Pontifical Catholic University of Peru, Lima, Peru; ^24^University of the Republic Uruguay, Montevideo, Uruguay; ^25^Instituto Universitário de Lisboa (ISCTE-IUL), CIS-IUL, Lisbon, Portugal; ^26^National Autonomous University of Mexico, ENES, Morelia, Mexico; ^27^University Setif 2, Setif, Algeria; ^28^University of Zagreb, Zagreb, Croatia; ^29^Chinese University of Hong Kong, Hong Kong, China; ^30^University of Malaya, Kuala Lumpur, Malaysia; ^31^Central University of Finance and Economics, Beijing, China; ^32^Aristotle University of Thessaloniki, Thessaloniki, Greece; ^33^University of Ljubljana, Ljubljana, Slovenia; ^34^University of Amsterdam, Amsterdam, Netherlands; ^35^University of Pécs, Pécs, Hungary; ^36^Università Cattolica del Sacro Cuore, Milan, Italy; ^37^Middle East University, Amman, Jordan; ^38^University of Granada, Granada, Spain; ^39^University of Delhi, New Delhi, India; ^40^VNU University of Social Sciences and Humanities, Vietnam National University, Hanoi, Vietnam; ^41^University of Havana, Havana, Cuba; ^42^University of Maribor, Maribor, Slovenia; ^43^Pontifical Catholic University of Rio de Janeiro, Rio de Janeiro, Brazil; ^44^Free University of Tbilisi, Tbilisi, Georgia; ^45^University of Vienna, Vienna, Austria; ^46^University Utara Malaysia, Sintok, Malaysia; ^47^İstanbul University, İstanbul, Turkey; ^48^Vilnius University, Vilnius, Lithuania; ^49^University of British Columbia, Vancouver, BC, Canada; ^50^Ivan Franko National University of Lviv, Lviv, Ukraine; ^51^University of Alberta, Edmonton, AB, Canada; ^52^Department of Psychology, Romanian Academy, UNATC-CINETIc Bucharest, Bucharest, Romania; ^53^Comenius University, Bratislava, Slovakia; ^54^Slovak Academy of Sciences, Bratislava, Slovakia; ^55^The Delve Pvt Ltd, Islamabad, Pakistan; ^56^School of Psychology, University of Monterrey, Nuevo Leon, Mexico; ^57^Aga Khan University Hospital, Karachi, Pakistan; ^58^Vivekananda College, University of Delhi, New Delhi, India; ^59^Pontifical Catholic University of Chile, Santiago, Chile; ^60^Universidad Latina de Costa Rica, San José, Costa Rica; ^61^Kyung Hee University, Seoul, South Korea; ^62^South-West University “Neofit Rilski”, Blagoevgrad, Bulgaria; ^63^Bahria University, Karachi, Pakistan

**Keywords:** cross-cultural, Facebook, motives, online social network, sex differences, age differences

## Introduction

Seventy-nine percent of internet users use Facebook, and on average they access Facebook eight times a day (Greenwood et al., 2016). To put these numbers into perspective, according to Clement (2019), around 30% of the world's population uses this Online Social Network (OSN) site.

Despite the constantly growing body of academic research on Facebook (Chou et al., 2009; Back et al., 2010; Kaplan and Haenlein, 2010; McAndrew and Jeong, 2012; Wilson et al., 2012; Krasnova et al., 2017), there remains limited research regarding the motivation behind Facebook use across different cultures. Our main goal was to collect data from a large cross-cultural sample of Facebook users to examine the roles of sex, age, and, most importantly, cultural differences underlying Facebook use.

### Cultural Differences

According to Clement (2019), the current total number of active monthly Facebook users is ~2.45 billion, including 183 million from the USA and 307 million from Europe, together constituting only 2% of the total number of Facebook users. Nevertheless, most previous research has focused on these two populations (e.g., USA: Ellison et al., 2006, 2007; Lampe et al., 2006; Stern et al., 2007; Raacke and Bonds-Raacke, 2008; Sheldon, 2008; Ophus and Abbitt, 2009; Pempek et al., 2009; Valenzuela et al., 2009; Smock et al., 2011; Europe: Joinson, 2008; Brandtzæg and Heim, 2009; Madge et al., 2009; Selwyn, 2009). This leaves the remaining 98% of the world's population of Facebook users almost unattended.

Among a few noteworthy exceptions, Abbas and Mesch (2015) investigated the role of cultural values in motivations for using Facebook among Palestinian youth in Israel. Błachnio et al. (2016) explored cultural and personality determinants of Facebook intrusion among eight countries from three continents. Jackson and Wang (2013) compared American and Chinese societies on the use of social networking sites. Finally, Ji et al. (2010) examined the use of social network services across American, Korean, and Chinese populations.

It is, thus, not surprising that many scholars stress the importance of cultural differences with regard to Facebook usage (Nadkarni and Hofmann, 2012; Wilson et al., 2012; Hsu et al., 2015), as cultural norms affect one's behavior in the context of online communication (Gevorgyan and Manucharova, 2009). For instance, people from individualistic cultures more often seek out information on social media sites, while people from collectivistic cultures tend to use social media more with an aim to obtain emotional support (Kim et al., 2011; USA & South Korea). The results of a study by Hsu et al. (2015; Australia, Austria, Japan, Taiwan, and USA) provided similar results, highlighting a major role of socialization and self-presentation in Facebook use among users from collectivist cultures.

Not only do scientists acknowledge the likely existence of cross-cultural differences in general motivations to use Facebook among distant and distinctive cultures (Wilson et al., 2012), studies also report differences in Facebook behaviors between seemingly similar countries (e.g., Strayhorn, 2009; Tsoi and Chen, 2011; Nadkarni and Hofmann, 2012). For example, people using Facebook in the UK value group-related content more than American users do, whereas Italian users rate groups and games as their most important online activities. It has also been reported that Greek Facebook users are less concerned with status updates in comparison with British, Italian, American, and French users (Vasalou et al., 2010). French users rate the motive of keeping in touch as more important than do Hong Kong users, while Hong Kong users display stronger preferences for communication, seeking and sharing information through Facebook (Tsoi and Chen, 2011). Finally, ethnic minorities appear to use Facebook more often than Caucasians do (for a review see: Nadkarni and Hofmann, 2012); scholars of a non-Caucasian origin also use Online Social Networks more frequently than their Caucasian counterparts (Strayhorn, 2009).

### Sex Differences

Are there sex differences in Facebook usage? According to Clement (2019), 54% of Facebook users declare to be a woman. Research conducted by Lin and Lu (2011; Taiwan) showed that the key factors for men's Facebook usage are “usefulness” and “enjoyment.” Women, on the other hand, appear more susceptible to peer influence. This is concurrent with the findings of Muise et al. (2009; Canada), in which longer times spent on Facebook correlated with more frequent episodes of jealousy-related behaviors and feelings of envy among women, but not men. Similarly, in Denti et al. (2012), Swedish women who spent more time on Facebook reported feeling less happy and less content with their life; this relationship was not observed among men.

In general, women tend to have larger Facebook networks (Stefanone et al., 2010; USA), and engage in more Facebook activities than men do (McAndrew and Jeong, 2012; USA; but see Smock et al., 2011; USA, who reported that women use Facebook chat less frequently than men). Another study (Makashvili et al., 2013; Georgia) provided evidence that women exceed men in Facebook usage due to their stronger desire to maintain contact with friends and share photographs, while men more frequently use Facebook to pass time and build new relationships.

### Age Differences

Early research showed that younger Facebook users tend to spend more time on Online Social Networks, and have a greater number of Facebook friends compared with older users (Joinson, 2008; UK). At the same time, young people are more likely to increase their profile's privacy. This may be due to the fact that older users have a relatively limited number of friends on their online friends list, typically invited, or accepted more carefully, thus, their need to secure their privacy is lower than among young users, who have a wider variety of friends, some of whom they met online (Dhir et al., 2017). This course of explanation is in line with Quinn et al. (2011; UK), who provided evidence that younger users (aged 15–30) have on average 11 times more Facebook friends than do older users (aged 50+).

Despite the fact that not all Facebook users are adolescents or young adults, most studies to date have been conducted specifically among such populations, leaving more mature users unattended (Manzi et al., 2018; Italy, Chile). Among the few exceptions are the studies of Oleszkiewicz et al. (2017), who examined emoticon usage on a large, diverse sample; Ancu (2012), who investigated motives for Facebook usage among American elders (50+); Newman et al. (2019), who attempted to develop a measurement of social network site use in older adults (UK); and Rattanasimakul (2015), who compared motivations and gratifications of Facebook use among three age groups (Thailand). Thus, building on the aforementioned research, one of our goals was to examine Facebook activities among different age cohorts (including middle-aged and senior adults).

### The Current Data

Conducting studies within only a limited number of countries or only within “Western” countries limits general conclusions about online social networking, as culture is an important predictor of various aspects of human behavior, including Facebook use (Nadkarni and Hofmann, 2012; Wilson et al., 2012; Hsu et al., 2015). To fill this gap, we aimed to investigate differences in reasons for Facebook usage (i.e., individual activities that draw people to use Facebook), using a large-scale cross-cultural sample (examining also sex and age of our participants).

## Materials and Methods

### Participants

A total of 16,465 individuals from 46 countries (68 study sites) participated in this research. Of these, 11,765 participants reported using Facebook and thus were included in analyses (see Table 1), whereas 4,700 (28.5%) were excluded from further analysis as individuals who did not meet the study criteria (i.e., do not use Facebook), or did not complete the questionnaire.

**Table 1:** Country, study sites, number of participants from each country, mean age (with SD), and sex ratio.

**Country**	**Study sites**	**Participants**	**Mean age (*SD*)**	**Sex ratio (% males)**
**Africa**		**895**	**25.69 (*****SD*** **=** **6.56)**	**462 (52%)**
Algeria	Algiers; Setif	431	26.33 (*SD* = 6.73)	197 (45%)
Nigeria	Nsukka	272	25.31 (*SD* = 5.82)	143 (53%)
Uganda	Gulu; Gampala	192	24.80 (*SD* = 7.01)	122 (64%)
**Asia**		**2,362**	**26.24 (*****SD*** **=** **8.26)**	**1,015 (43%)**
China	Beijing	192	26.12 (*SD* = 9.02)	93 (48%)
India	Delhi	304	25.41 (*SD* = 8.88)	162 (53%)
Indonesia	Jakarta; Yogyakarta	68	20.05 (*SD* = 2.27)	26 (38%)
Iran	Shiraz	112	25.80 (*SD* = 6.71)	52 (46%)
Jordan	Ma'an	166	21.56 (*SD* = 4.26)	92 (55%)
Malaysia	Sintok; Kuala Lumpur	179	29.62 (*SD* = 10.38)	80 (44%)
Pakistan	Iahore; Islamabad; Karachi	606	26.75 (*SD* = 8.42)	245 (40%)
Russia	Moscow; Ussuriisk	104	27.41 (*SD* = 8.91)	39 (37%)
South Korea	Seoul	141	26.35 (*SD* = 9.57)	79 (56%)
Vietnam	Hanoi	489	27.20 (*SD* = 6.47)	146 (29%)
**Australia**	Canberra	**425**	**27.58 (*****SD*** **=** **9.02)**	**202 (48%)**
**Europe**		**6,281**	**27.02 (*****SD*** **=** **9.32)**	**2,826 (45%)**
Austria	Vienna	160	23.46 (*SD* = 6.15)	37 (23%)
Belgium	Louvain la Neuve	372	28.68 (*SD* = 8.80)	167 (44%)
Bulgaria	Blagoevgrad; Sofia	113	23.89 (*SD* = 7.87)	41 (36%)
Croatia	Zagreb	299	24.63 (*SD* = 9.37)	135 (45%)
Estonia	Tartu	182	26.04 (*SD* = 7.89)	83 (45%)
Georgia	Tbilisi	189	27.66 (*SD* = 10.88)	93 (49%)
Germany	Dresden	79	26.76 (*SD* = 6.99)	26 (26%)
Greece	Crete; Athens	115	29.68 (*SD* = 11.25)	43 (37%)
Hungary	Budapest; Pecs; Várpalota; Székesfehérvár; Zalaegerszeg	779	28.66 (*SD* = 10.26)	379 (48%)
Italy	Milan	325	27.42 (*SD* = 10.55)	104 (32%)
Lithuania	Vilnius	259	26.53 (*SD* = 8.63)	128 (49%)
Netherlands	Amsterdam	177	28.92 (*SD* = 11.44)	65 (36%)
Norway	Trondheim	267	23.20 (*SD* = 2.82)	143 (53%)
Poland	Brzeg; Gdansk; Wrocław	448	25.68 (*SD* = 7.25)	252 (56%)
Portugal	Lisbon	246	26.52 (*SD* = 9.11)	97 (39%)
Romania	Bucharest	170	25.99 (*SD* = 8.97)	84 (49%)
Slovakia	Trnava; Nitra	126	26.95 (*SD* = 7.54)	51 (40%)
Slovenia	Ljubljana; Maribor	490	27.00 (*SD* = 8.71)	236 (48%)
Spain	Granada	182	23.53 (*SD* = 4.24)	58 (31%)
Sweden	Stockholm	277	28.29 (*SD* = 10.37)	133 (48%)
Turkey	Istanbul; Izmir	813	28.97 (*SD* = 10.71)	369 (45%)
Ukraine	Lviv	214	26.14 (*SD* = 8.46)	103 (48%)
**Latin America**		**1,602**	**27.55 (*****SD*** **=** **10.67)**	**672 (43%)**
Brazil	Rio de Janeiro; São Paulo	279	28.77 (*SD* = 11.57)	126 (45%)
Chile	Santiago	176	31.17 (*SD* = 13.14)	72 (40%)
Colombia	Bogotá; Cucuta; Tunja	170	27.82 (*SD* = 11.67)	78 (45%)
Costa Rica	San José	121	37.61 (*SD* = 8.01)	61 (50%)
Cuba	Havana; Vereda Nueva; Consolación del Sur	123	24.59 (*SD* = 8.26)	49 (39%)
El Salvador	San Salvador	86	24.10 (*SD* = 7.46)	31 (36%)
Mexico	Mexico city	163	25.50 (*SD* = 7.71)	67 (32%)
Peru	Lima	249	21.88 (*SD* = 6.40)	92 (36%)
Uruguay	Montevideo	235	28.39 (*SD* = 10.10)	96 (40%)
**North America**		**200**	**20.75 (*****SD*** **=** **4.35)**	**74 (37%)**
United States	Austin	200	20.75 (*SD* = 4.35)	74 (37%)
**In total**		**11,765**	**26.75 (*****SD*** **=** **9.10)**	**5,251 (45%)**

*Bolded items represent sums for each continent and for the entire dataset*.

In order to create as diverse and yet comparable samples from each country as possible, members of the research team were instructed to recruit half of their participants from samples of local students, and the other half from the local community at large. From study sites that kept records of the source of their samples (22 countries), 47.14% of participants came from community samples. We exercised great care to ensure similar recruitment methods in all study sites, which included poster adverts, emails, and word-of-mouth. Participants were not compensated for their participation in the study.

### Procedure

Prior to data collection, the corresponding author discussed the questionnaire with all the collaborating research groups. In those countries where English was not a first or primary language, participants could complete the questionnaire in their native language. This procedure involved translating the measures from English into the native language, and then, by a different collaborator, back-translating the questionnaire items into English. Any differences in translated versions were then discussed until an agreement was made on the most appropriate translation (Brislin, 1970). If there was more than one study site from each country, all local groups were asked to participate in the translation process.

As internet surveys tend to be under-representative, especially in developing countries (Batres and Perrett, 2014), all data were collected in person by a network of research teams. The instructions for participants were as follows: “You are being asked to participate in an anonymous survey study. Your participation is entirely voluntary. This survey was designed to compare various variables and constructs around the world—it will be completed by participants in 40 countries. Please remember that there are no right or wrong answers in this survey (what matters are your opinions). If you wish to participate, please continue with the questionnaire. If not, please do not complete the questionnaire” (English version). After providing informed, written consent to participate in the study, participants were given a set of questionnaires, including the current Facebook scale, and several unrelated questionnaires in the context of a broader cross-cultural research project (see e.g., Conroy-Beam et al., 2019a,b). (1) The instructions for completing the Facebook scale were as follows: “Do you use Facebook? Yes/No (if no, please continue to the next scale); (2) Please use the scale below (ranging from 1—very rarely, to 5—very often) to assess how often you use Facebook for the following purposes” (see *Measures* below). Participants completed questionnaires in ~30 min. Data were collected simultaneously across all locations, and then coded, standardized, and merged into one dataset.

### Measures

We examined individual reasons for Facebook usage using a 13-item scale, constructed for the purpose of this study and based on previous research (Steinfield et al., 2008; Vasalou et al., 2010; Wilson et al., 2012; Krasnova et al., 2017). The scale listed the most common and recurring reasons for Facebook use: to keep in touch with friends, reconnect with people with whom one has lost contact, relieve boredom, organize or join events, join groups, to present one's opinions and beliefs, see what friends are doing, inform others of what one is doing, post and share photos, write private messages, make new friends, date new people, and look at the profiles of people one does not know (see https://figshare.com/s/68f306f31958d9a5c0c0, for English version of the scale). Participants reported how often they use Facebook for each of the 13 aforementioned reasons (on a Likert scale, ranging from 1—*very rarely*, to 5—*very often*). The questionnaire also included basic socioeconomic measures (e.g., sex, age, material situation, see database under the link: https://figshare.com/s/68f306f31958d9a5c0c0).

### Strengths and Limitations

The present dataset has several strengths, which distinguish it from other studies: (1) it was conducted on a large number of participants (*N* = 11,765); (2) we considered six different regions of the world (Africa, Asia, Australia, Europe, Latin America, and North America), some of which have only been included in a handful of previous studies (e.g., Australia & Asia: Hsu et al., 2015; Asia: Kim et al., 2011; Makashvili et al., 2013); (3) all participants filled in the same questionnaires; (4) all persons took part in the study in the same years; (5) we measured additional variables, which may be useful in further analyses, replications and extensions of the current research (i.e., sex, age, education level, years of study, economic situation, and religious affiliation).

Despite these contributions, the present dataset has several limitations that hinder drawing general conclusions. First, the study samples from each of the study sites are not representative for the whole of each country, as half of the participants were students, and the other half originated from the local community; some sub-populations or cultural groups may have been excluded. Second, our questionnaire did not include any items related to entertainment (for instance, games), which is a significant omission, as playing games on Facebook has been shown to be a popular activity (Wohn and Lee, 2013). Third, as our questionnaire consisted of one-item questions regarding various reasons for using Facebook, a reliability analysis could not be performed. Last, but not least, we have not collected data on participants' frequency of Facebook use, Facebook membership duration, and their number of Facebook friends. These variables were previously linked to Facebook usage, so a lack of such data in the present dataset leaves open directions for future research.

### Possible Research Paths

Based on the presented dataset, scholars can conduct numerous analyses concerning a broad range of research questions. They can explore reasons for Facebook usage in various cultures, with regard to, for instance, age, sex, country of origin, culture, and numerous other variables. Preliminary analyses (see: https://figshare.com/s/68f306f31958d9a5c0c0) presented statistically significant differences in the range of all identified, individual reasons for Facebook use across continents (*p* < 0.001), but the effect sizes for the given differences were rather small (ranging from 0.01 to 0.10). The highest effect size was observed in presenting opinions (*d* = 0.102). Furthermore, the analysis indicated that there are significant sex differences in all reported reasons for using Facebook, except for presenting opinions and writing private messages. However, the effect sizes for sex differences were small (from dating, *d* = 0.211, higher among men; to reducing the sense of boredom, *d* = −0.147; higher among women). Additionally, there were statistically significant but generally weak relationships between age and several of the reported reasons behind Facebook usage. Nevertheless, age explained <1% of the variation in any declared purpose for Facebook usage. In future analyses, the present dataset and aforementioned differences could also be investigated on a cross-cultural level.

In addition, scientists may apply these data to identify other country-level predictors of Facebook usage. For example, the degree of Facebook usage: degree of individualistic/collectivistic values preferred within a given culture, which can be easily obtained from other online sources (e.g., Hofstede's culture dimensions: Hofstede, 2001); Schwartz's value orientations (Schwartz, 2006); degree of Westernization (Gunewardene et al., 2001); or power distance (Hofstede, 2006). Also, as previous research has offered conflicting results regarding the differences (or lack thereof) between different cultures in Facebook usage (Karl et al., 2010; Manzi et al., 2018), the data presented herein (conducted on a large sample across the world) may serve as a reference point for further investigations regarding Online Social Media usage.

### Dataset Description

The data in the present paper have been deposited in the Figshare repository and are freely accessible through the following link: https://figshare.com/s/68f306f31958d9a5c0c0 under the name: “Reasons for Facebook usage: data from 46 countries.” The deposit contains five files: Radme_facebook, containing the basic information regarding the present data, Codebook_facebook, containing definitions of variables and values, along with a short summary of each variable, Database_facebook, containing the raw data, Facebook_questionnaire, containing the English version of the questionnaire, and Preliminary_analysis, containing the comparison regarding sex, age, and cultures (grouped within continents).

## Data Availability Statement

All datasets generated for this study are included in the article/Figshare, under the link: https://figshare.com/s/68f306f31958d9a5c0c0.

## Ethics Statement

This study was carried out in accordance with the recommendations of the Institutional Review Board of the University of Wroclaw, and the Declaration of Helsinki. The Institutional Review Board of the Institute of Psychology at the University of Wroclaw gave ethical approval for conducting the study. All participants provided written informed consent to participate in the study.

## Author Contributions

All authors listed have made substantial, direct and intellectual contribution to the work, and approved it for publication. MK, PS, MD, KP, AO, CAl, JC-G, LK, MBu, and PP contributed to the preparation of the manuscript. PS and AS coordinated the project. Remaining authors collected the data.

## Conflict of Interest

MR was employed by company The Delve Pvt Ltd, Islamabad, Pakistan. The remaining authors declare that the research was conducted in the absence of any commercial or financial relationships that could be construed as a potential conflict of interest.

## References

[B1] Abbas R. Mesch G. S. (2015). Cultural values and facebook use among Palestinian youth in Israel. Comput. Human Behav. 48, 644–653. 10.1016/j.chb.2015.02.031

[B2] Ancu M. (2012). Older adults on Facebook: a survey examination of motives and use of social networking by people 50 and older. Fla. Commun. J. 40 1–12.

[B3] Back M. D. Stopfer J. M. Vazire S. Gaddis S. Schmukle S. C. Egloff B.. (2010). Facebook profiles reflect actual personality, not self-idealization. Psychol. Sci. 21, 372–374. 10.1177/0956797609360756 20424071

[B4] Batres C. Perrett D. I. (2014). The influence of the digital divide on face preferences in El Salvador: People without internet access prefer more feminine men, more masculine women, and women with higher adiposity. PLoS One 9:e100966 10.1371/journal.pone.0100966 25006801 PMC4089996

[B5] Błachnio A. Przepiorka A. Benvenuti M. Cannata D. Ciobanu A. M. Senol-Durak E.. (2016). Cultural and personality predictors of facebook intrusion: a cross-cultural study. Front. Psychol. 7:1895. 10.3389/fpsyg.2016.01895 27994566 PMC5134356

[B6] Brandtzæg P. B. Heim J. (2009). Why people use social networking sites, in Online Communities and Social Computing, eds Ozok A. A. Zaphiris P. (Berlin: Springer), 143–152.

[B7] Brislin R. W. (1970). Back-translation for cross-cultural research. J. Cross Cult. Psychol. 1, 185–216.

[B8] Chou W. S. Hunt Y. M. Beckjord E. B. Moser R. P. Hesse B. W. (2009). Social media use in the United States: implications for health communication. J. Med. Internet Res. 11:e48. 10.2196/jmir.1249 19945947 PMC2802563

[B9] Clement J. (2019). Number of Monthly Active Facebook Users Worldwide as of 4th Quarter 2019 (in Millions). Facebook Statistics. Retrieved from: https://www.statista.com/statistics/264810/number-of-monthly-active-facebook-users-worldwide/ (accessed February 15, 2020).

[B10] Conroy-Beam D. Buss D. M. Asao K. Sorokowska A. Sorokowski P. Aavik T.. (2019a). Contrasting computational models of mate preference integration across 45 Countries. Sci. Rep. 9, 1–13. 10.1038/s41598-019-52748-8 31729413 PMC6858324

[B11] Conroy-Beam D. Roney J. R. Lukaszewski A. W. Buss D. M. Asao K. Sorokowska A. (2019b). Assortative mating and the evolution of desirability covariation. Evol. Hum. Behav. 40, 479–491. 10.1016/j.evolhumbehav.2019.06.003

[B12] Denti L. Nilsson I. Barbopoulos I. Holmberg L. Thulin M. Wendeblad M. (2012). Sweden's Largest Facebook Study: A Survey of 1000 Swedish Facebook Users. Gothenburg: Gothenburg Research Institute.

[B13] Dhir A. Torsheim T. Pallesen S. Andreassen C. S. (2017). Do online privacy concerns predict selfie behavior among adolescents, young adults and adults? Front. Psychol. 8:815. 10.3389/fpsyg.2017.00815 28588530 PMC5440591

[B14] Ellison N. Steinfield C. Lampe C. (2006). Spatially bounded online social networks and social capital: the role of Facebook, in Presented at the Annual Conference of the International Communication Association (Dresden).

[B15] Ellison N. B. Steinfield C. Lampe C. (2007). The benefits of facebook “Friends:” social capital and college students' use of online social network sites. J. Comput. Mediat. Commun. 12, 1143–1168. 10.1111/j.1083-6101.2007.00367.x

[B16] Gevorgyan G. Manucharova N. (2009). Does culturally adapted online communication work? a study of american and chinese internet users' attitudes and preferences toward culturally customized web design elements. J. Comput. Mediat. Commun. 14, 393–413. 10.1111/j.1083-6101.2009.01446.x

[B17] Greenwood S. Perrin A. Duggan M. (2016). Social Media Update 2016. Pew Research Center, 11.

[B18] Gunewardene A. Huon G. F. Zheng R. (2001). Exposure to westernization and dieting: a cross-cultural study. Int. J. Eat. Disord. 29, 289–293. 11262507 10.1002/eat.1020

[B19] Hofstede G. (2001). Culture's Consequences: Comparing Values, Behaviors, Institutions and Organizations Across Nations. London: Sage Publications.

[B20] Hofstede G. (2006). What did GLOBE really measure? Researchers' minds versus respondents' minds. J. Int. Bus. Stud. 37, 882–896. 10.1057/palgrave.jibs.8400233

[B21] Hsu M. H. Tien S. W. Lin H. C. Chang C. M. (2015). Understanding the roles of cultural differences and socio-economic status in social media continuance intention. Inf. Technol. People 28, 224–241. 10.1108/ITP-01-2014-0007

[B22] Jackson L. A. Wang J.-L. (2013). Cultural differences in social networking site use: a comparative study of China and the United States. Comput. Human. Behav. 29, 910–921. 10.1016/j.chb.2012.11.024

[B23] Ji Y. G. Hwangbo H. Yi J. S. Rau P. L. P. Fang X. Ling C. (2010). The influence of cultural differences on the use of social network services and the formation of social capital. Int. J. Hum. Comput. Interact. 26, 1100–1121. 10.1080/10447318.2010.516727

[B24] Joinson A. N. (2008). ‘Looking at’, ‘looking up’ or ‘keeping up with’ people? motives and uses of facebook. Online Soc. Net. 10, 1027–1036. 10.1145/1357054.1357213

[B25] Kaplan A. M. Haenlein M. (2010). Users of the world, unite! The challenges and opportunities of Social Media. Bus. Horiz. 53, 59–68. 10.1016/j.bushor.2009.09.003

[B26] Karl K. Peluchette J. Schlaegel C. (2010). Who's posting facebook faux pas? a cross-cultural examination of personality differences. Int. J. Sel. Assess. 18, 174–186. 10.1111/j.1468-2389.2010.00499.x

[B27] Kim Y. Sohn D. Choi S. M. (2011). Cultural difference in motivations for using social network sites: a comparative study of American and Korean college students. Comput. Human Behav. 27, 365–372. 10.1016/j.chb.2010.08.015

[B28] Krasnova H. Veltri N. F. Eling N. Buxmann P. (2017). Why men and women continue to use social networking sites: The role of gender differences. J. Strategic Inf. Sys. 26, 261–284. 10.1016/j.jsis.2017.01.004

[B29] Lampe C. Ellison N. Steinfield C. (2006). A face(book) in the crowd: social searching vs. social browsing, in Proceedings of the 2006 20th Anniversary Conference on Computer Supported Cooperative Work - CSCW '06 (Banff). 167.

[B30] Lin K.-Y. Lu H.-P. (2011). Why people use social networking sites: an empirical study integrating network externalities and motivation theory. Comput. Human Behav. 27, 1152–1161. 10.1016/j.chb.2010.12.009

[B31] Madge C. Meek J. Wellens J. Hooley T. (2009). Facebook, social integration and informal learning at university: ‘it is more for socialising and talking to friends about work than for actually doing work.’ Learn. Media Technol. 34, 141–155. 10.1080/17439880902923606

[B32] Makashvili M. Ujmajuridze B. Amirejibi T. Kotetishvili B. Barbakadze S. (2013). Gender difference in the motives for the use of facebook. Asian J. Res. Soc. Sci. Humanit. 01, 130–135. 10.13140/2.1.4946.7845

[B33] Manzi C. Coen S. Regalia C. Yévenes A. M. Giuliani C. Vignoles V. L. (2018). Being in the social: a cross-cultural and cross-generational study on identity processes related to facebook use. Comput. Human Behav, 80, 81–87. 10.1016/j.chb.2017.10.046

[B34] McAndrew F. T. Jeong H. S. (2012). Who does what on facebook? age, sex, and relationship status as predictors of facebook use. Comput. Human Behav. 28, 2359–2365. 10.1016/j.chb.2012.07.007

[B35] Muise A. Christofides E. Desmarais S. (2009). More information than you ever wanted: does facebook bring out the green-eyed monster of jealousy? CyberPsychol. Behav. 12, 441–444. 10.1089/cpb.2008.0263 19366318

[B36] Nadkarni A. Hofmann S. G. (2012). Why do people use Facebook? J. Individ. Differ. 52, 243–249. 10.1016/j.paid.2011.11.007 22544987 PMC3335399

[B37] Newman L. Stoner C. Corbett A. Megalogeni M. Khan Z. Spector A. (2019). Development of the ‘SNS older adults measure’ (SNS-OA) to examine social network site use in older adults. Aging Ment. Health 11, 1–10. 10.1080/13607863.2019.1673700 31603018

[B38] Oleszkiewicz A. Karwowski M. Pisanski K. Sorokowski P. Sobrado B. Sorokowska A. (2017). Who uses emoticons? data from 86 702 facebook users. Pers. Individ. Dif. 119, 289–295. 10.1016/j.paid.2017.07.034

[B39] Ophus J. D. Abbitt J. T. (2009). Exploring the potential perceptions of social networking systems in university courses. J. Online Learn. Teach. 5, 639–648.

[B40] Pempek T. A. Yermolayeva Y. A. Calvert S. L. (2009). College students' social networking experiences on Facebook. J. Appl. Dev. Psychol. 30, 227–238. 10.1016/j.appdev.2008.12.010

[B41] Quinn D. Chen L. Mulvenna M. (2011). Does age make a difference in the behaviour of online social network users?, in 2011 IEEE International Conference on Internet of Things and 4th IEEE International Conference on Cyber, Physical and Social Computing (IThings/CPSCom 2011) (Dalian), 266–272.

[B42] Raacke J. Bonds-Raacke J. (2008). MySpace and facebook: applying the uses and gratifications theory to exploring friend-networking sites. CyberPsychol. Behav. 11, 169–174. 10.1089/cpb.2007.0056 18422409

[B43] Rattanasimakul K. (2015). The uses and gratification of facebook among adolescents, working ages and elderly. J. Public Relat. Advert. 8, 14–20.

[B44] Schwartz S. (2006). A theory of cultural value orientations: explication and applications. Comp. Sociol. 5, 137–182.

[B45] Selwyn N. (2009). Faceworking: exploring students' education-related use of Facebook. Learn. Media Technol. 34, 157–174. 10.1080/17439880902923622

[B46] Sheldon P. (2008). The relationship between unwillingness-to-communicate and students' facebook use. J. Media Psychol. 20, 67–75. 10.1027/1864-1105.20.2.67

[B47] Smock A. D. Ellison N. B. Lampe C. Wohn D. Y. (2011). Facebook as a toolkit: a uses and gratification approach to unbundling feature use. Comput. Human Behav. 27, 2322–2329. 10.1016/j.chb.2011.07.011

[B48] Stefanone M. A. Lackaff D. Rosen D. (2010). Contingencies of self-worth and social-networking-site behavior. Cyberpsychol. Behav. Soc. Net. 14, 41–49. 10.1089/cyber.2010.0049 21329442

[B49] Steinfield C. Ellison N. B. Lampe C. (2008). Social capital, self-esteem, and use of online social network sites: a longitudinal analysis. J. Appl. Dev. Psychol. 29, 434–445. 10.1016/j.appdev.2008.07.002

[B50] Stern L. A. Taylor K. Yergensen B. Tyma A. Rahoi-gilchrest R. L. Charlesworth D. (2007). Communication, speech & theatre association of north Dakota editor. J. Commun. Speech Theatre Assoc. North Dakota 20, 9–20.

[B51] Strayhorn T. L. (2009). An examination of the impact of first-year seminars on correlates of college student retention. J. First Year Exp. Stud. Transit. 21, 9–27.

[B52] Tsoi H. K. Chen L. (2011). From privacy concern to uses of social network sites: a cultural comparison via user Survey, in 2011 IEEE Third International Conference on Privacy, Security, Risk and Trust and 2011 IEEE Third International Conference on Social Computing (Boston, MA), 457–464.

[B53] Valenzuela S. Park N. Kee K. F. (2009). Is there social capital in a social network site?: facebook use and college students' life satisfaction, trust, and participation. J. Comput. Mediat. Commun. 14, 875–901. 10.1111/j.1083-6101.2009.01474.x

[B54] Vasalou A. Joinson A. N. Courvoisier D. (2010). Cultural differences, experience with social networks and the nature of “true commitment” in Facebook. Int. J. Human Comput. Stud. 68, 719–728. 10.1016/j.ijhcs.2010.06.002

[B55] Wilson R. E. Gosling S. D. Graham L. T. (2012). A review of facebook research in the social sciences. Perspect. Psychol. Sci. 7, 203–220. 10.1177/1745691612442904 26168459

[B56] Wohn D. Y. Lee Y. H. (2013). Players of Facebook games and how they play. Entertain. Comput. 4, 171–178. 10.1016/j.entcom.2013.05.002

